# Spatial point analysis based on dengue surveys at household level in central Brazil

**DOI:** 10.1186/1471-2458-8-361

**Published:** 2008-10-20

**Authors:** João B Siqueira-Junior, Ivan J Maciel, Christovam Barcellos, Wayner V Souza, Marilia S Carvalho, Nazareth E Nascimento, Renato M Oliveira, Otaliba Morais-Neto, Celina MT Martelli

**Affiliations:** 1 Institute of Tropical Pathology and Public Health, Federal University of Goias, Department of Collective Health, Goias, Brazil; 2 Oswaldo Cruz Foundation, DIS/CICT, Rio de Janeiro, Brazil; 3 Oswaldo Cruz Foundation, Centro de Pesquisas Aggeu Magalhães, Pernambuco, Brazil; 4 Scientific Computation Program, Oswaldo Cruz Foundation, Rio de Janeiro, Brazil

## Abstract

**Background:**

Dengue virus (DENV) affects nonimunne human populations in tropical and subtropical regions. In the Americas, dengue has drastically increased in the last two decades and Brazil is considered one of the most affected countries. The high frequency of asymptomatic infection makes difficult to estimate prevalence of infection using registered cases and to locate high risk intra-urban area at population level. The goal of this spatial point analysis was to identify potential high-risk intra-urban areas of dengue, using data collected at household level from surveys.

**Methods:**

Two household surveys took place in the city of Goiania (~1.1 million population), Central Brazil in the year 2001 and 2002. First survey screened 1,586 asymptomatic individuals older than 5 years of age. Second survey 2,906 asymptomatic volunteers, same age-groups, were selected by multistage sampling (census tracts; blocks; households) using available digital maps. Sera from participants were tested by dengue virus-specific IgM/IgG by EIA. A Generalized Additive Model (GAM) was used to detect the spatial varying risk over the region. Initially without any fixed covariates, to depict the overall risk map, followed by a model including the main covariates and the year, where the resulting maps show the risk associated with living place, controlled for the individual risk factors. This method has the advantage to generate smoothed risk factors maps, adjusted by socio-demographic covariates.

**Results:**

The prevalence of antibody against dengue infection was 37.3% (95%CI [35.5–39.1]) in the year 2002; 7.8% increase in one-year interval. The spatial variation in risk of dengue infection significantly changed when comparing 2001 with 2002, (ORadjusted = 1.35; p < 0.001), while controlling for potential confounders using GAM model. Also increasing age and low education levels were associated with dengue infection.

**Conclusion:**

This study showed spatial heterogeneity in the risk areas of dengue when using a spatial multivariate approach in a short time interval. Data from household surveys pointed out that low prevalence areas in 2001 surveys shifted to high-risk area in consecutive year. This mapping of dengue risks should give insights for control interventions in urban areas.

## Background

The global impact of dengue fever has grown dramatically in recent decades reflecting the geographic dispersion of several vector species and the introduction or co-circulation of different dengue virus serotypes (DEN1-4) in susceptible human populations [1-3]. The rapid, often unplanned urban growth in many tropical and subtropical regions has created an appropriate environment for mosquito breeding sites due in part to problems with water supply, drainage and waste disposal. These factors, combined with increased mobility in the population and improved transportation infrastructure has the potential for sustaining inter- and intra-urban virus transmission, thereby increasing the importance of the dengue fever threat to the public's health in most of the Americas, Southeast Asia and Western Pacific countries [3-5].

Dengue transmission is determined mainly by the ecology of susceptible populations in the local environment, mosquito density, and the circulating serotype(s) of the virus [6,7]. Dengue serosurveys have been used previously to estimate the prevalence of dengue at the community level, to characterize the population at risk, and to assess individual and area-based factors associated with infection [8-11]. Some studies have provided geographic characteristics of disease incidence and prevalence aggregated by areal units, limited to some degree by the constraints of the zoning systems used to collect information, such as census tract [12,13]. It is well known that the process of viral diffusion is spatially continuous and thus not restricted by administrative boundaries [14]. In the last decade several studies have used geographic information systems to explore the distribution of dengue surveillance data and dispersion of viral serotypes and vector populations to better target intervention areas [6,12,15-17].

In Brazil, one of the countries in the Americas most affected by dengue fever, disease incidence in the population occurred initially as epidemic waves (1986–1993), followed thereafter an intense countrywide dengue virus circulation [18]. Approximately 1.3 million cases were reported during the epidemic years of 1998 and 2002. Currently, three serotypes (DENV1, 2 and 3) co-circulate in most areas of Brazil and an increasing trend in hospitalization rates has been observed, suggesting a shift in disease severity [18]. In a previous paper, we reported an overall 29.5% prevalence of antibodies against dengue virus in a household survey conducted in a densely populated urban area in Central Brazil in the year 2001 [10]. In this manuscript we described two serosurveys (2001 and 2002) using Generalized Additive Model (GAM) in order to depict the spatial risk distribution of dengue infection in urban area. We explored the spread of dengue infection in a city in Central Brazil, where the virus has been recently introduced.

## Methods

### Study area and population

Two surveys were conducted from January-February 2001 and in the same period in 2002 in the city of Goiania (~1.1 million inhab), Central Brazil. Since the virus introduction in the year 1994, dengue is part of the nationwide surveillance system as reportable disease, affecting predominantly among adults population. Local laboratory surveillance detected DEN-1 as the main circulating serotype, followed by DEN-2 from 1994 to the beginning of 2002 [19]. Details of the design and methodology of the first household survey have been previously described [10]. Briefly, during 2001 a total of 1,586 individuals older than 5 years of age were selected for interview, using a multistage sampling approach to achieve a representative sample of the municipality. The overall dengue prevalence rate among survey participants was 29.5%, based on dengue-specific immunoglobulin (IgM/IgG) testing of serum using enzyme-linked immunoassay (EIA). This survey showed estimated prevalence for the six administrative macro-regions ranging from 23.6 to 41.6% [10]. Estimated prevalence surface peaked at nearly 50% in outlying areas of the city based on spatial point pattern analysis using the dual Kernel method [10,14,20].

#### Survey 2002

A sample size of 3,000 participants was calculated based on the power needed to detect an estimated 5% increase in prevalence for each of the six macro-regions, taking into account the 2001 serosurvey results. A three stage sampling approach was applied to select participants in 2002. First, census tracts were sampled with probability proportional to the number of occupied houses (year 2000 census data) [21]; 50% of the total 1,066 census tracts were sampled using the available digital city map. Secondly, blocks and subsequently households in each tract were randomly selected and maps were plotted using the geocoded location of households to guide fieldwork (ArcView GIS software, version 3.2; Environmental Systems Research Institute, Inc., Redlands, United States). An additional 15% of total residences were plotted to replace any empty or closed buildings encountered during the survey. Thirdly, during the household visit, one resident aged 5 years or older was randomly selected and invited to participate in the survey by responding to a questionnaire and providing a blood sample. This sampling scheme allowed selecting approximately 450 individuals in each of six macro-regions, providing a comprehensive distribution of participants within the urban area. The survey was conducted during afternoon and evening hours, including weekends; the survey of the entire urban area was completed in 6 weeks.

#### Variables

In both surveys, a blood sample was taken and the serum tested by dengue virus-specific IgM/IgG by EIA (PANBIO^® ^INDX, INC, Baltimore, MD, USA); this commercial kit identifies antibody to all four dengue virus serotypes. The cut-off point for seropositive samples was established as ≥ 0.5 optical density (OD) units, and testing included positive and negative control sera in each plate for quality control and ascertainment of the expected cut-off values. Borderline were retested and yielded negative results. Positive results were considered infected cases and negative non-cases (outcome variable). Individual-level variables were collected during household visits included data on sex, age, education, self-reported and family history of dengue. The same questionnaire was applied in both surveys.

Each participant in the first survey was manually georeferenced by residential address into the available digital map of the city using ArcView software, generating a point pattern layer. In the second survey, we took advantage of the point pattern layer generated in advance of fieldwork which depicted the exact geocoded residential position of 100% of households visited. The data from both surveys were merged into a single database and the year of the survey was considered as an explanatory variable for the generalized additive model.

### Data analysis

The main characteristics of the sampled populations in the two serosurveys were compared using chi-square tests. We performed the descriptive and exploratory data analysis using SPSS software for Windows (version 10.0; SPSS Inc., Chicago, Illinois).

### Spatial Point Analysis

A Generalized Additive Model (GAM) is a statistical model that extends the generalized linear models to include non-parametric smoothing terms [22]. In the generalized linear model the response variable (Y) belongs to the exponential family, and its mean value is related to the linear predictors through a link function. We applied the link function logit (log(p/(1-p))) for binomial response, such as positive or negative sera.

In the case of GAM models the linear predictor η is a sum of terms including different types of covariates (χ_1_, χ_2_...), as in the linear model, and special smoothing terms, in our study latitude and longitude of the household of each participant: η_ι _= β_0_+β_1_χ_1_+β_2_χ_2_+...+*f*_1_(lat_i_, long_i_). The smoothing function used was a thin plate regression spline, that can be described as piece-wise polynomial functions that fit together at the points of inflexion [23]. Models were fitted in R, with mgcv library [24].

This procedure allowed estimating the probability of finding a positive event in any given cloud of sampling points. This spatial analysis was applied to generate smoothed values positive by total events, i.e., an estimate of area dengue infection relative risk. In short, the GAM output produces an adjusted map representing the spatial estimate odds ratio of infection.

#### Ethical issues

Permission to carry out both surveys was provided by the Ethical Review Committee of Federal University of Goias. Written informed consent was obtained for all participants and in case of minor, from their legal guardians.

## Results

The characteristics of the 1,586 participants of the 2001 survey, and those of the 2,906 participants during 2002 were presented in Table 1. The proportion of participants in both surveys was similar with respect to age distribution, gender, education and yellow fever vaccination status. In the first survey, the overall prevalence of antibody prevalence against dengue virus was 29.5% (95%CI [27.3–31.8%]) as described previously [10]. In the second survey, seroprevalence was 37.3% (95%CI [35.5–39.1]) corresponding to an estimated incidence of infection of 7.8% during the one-year interval. The proportion of infected individuals increased with age, and there was no difference in prevalence with respect to gender in each survey.

**Table 1:** Characteristics of the participants of two population-based dengue serosurveys, Central Brazil, 2001–2002

Characteristics	2001 Survey (n = 1,586)		2002 Survey (n = 2,906)	
	
	No	%	No	%
Gender:				
Female	1,066	67.2	1,967	67.7
Male	520	32.8	939	32.3
Age (years):				
5–14	63	4.0	133	4.6
15–49	1,084	68.3	1,969	67.8
≥ 50	438	27.6	794	27.3
Unknown	1	0.1	10	0.3
Educational Level				
Illiterate	99	6.2	182	6.3
Elementary school	920	58.0	1,643	56.5
High school	423	26.7	793	27.3
College	133	8.4	268	9.2
Unknown	11	0.7	20	0.7
Yellow fever vaccination history				
Yes	1,451	91.5	2,706	93.1
No	89	5.6	126	4.3
Unknown	46	2.9	74	2.5
Seropositives*	506	29.5†	1,144	37.3†

* Seropositive refers to IgM/IgG positive results for dengue virus infections.

† p < 0.01 (two sided; χ^2 ^test statistic comparing 2001 and 2002 serosurveys)

Table 2 shows the adjusted odds ratio for the covariates included in the GAM model. A significant increase in dengue prevalence was observed in the year 2002 compared to 2001 (OR = 1.35; 95%CI [1.18–1.54]). Prevalence of dengue infection increased with age and history of dengue in the family was also associated with the outcome. Education level had an inverse association with past dengue infection (p values < 0.001).

**Table 2:** Generalized Additive Model (GAM) results for serological evidence of past dengue infection in the city of Goiânia, Central Brazil.

Variables	Adjusted Odds Ratio (95% Confidence Interval)	p value
Year		
2001	1	
2002	1.35 (1.18–1.54)	< 0.001
Age group		
5–14	1	
15–49	2.29 (1.59–3.30)	< 0.001
50 – 99	3.08 (2.12–4.47)	< 0.001
Sex		
Male	1	
Female	1.01 (0.89–1.16)	0.83
Education		
University	1	
Secondary	1.56 (1.21–2.03)	< 0.001
Primary	1.96 (1.54–2.50)	< 0.001
Illiterate	2.72 (1.94–3.82)	< 0.001

**Figure 1:**
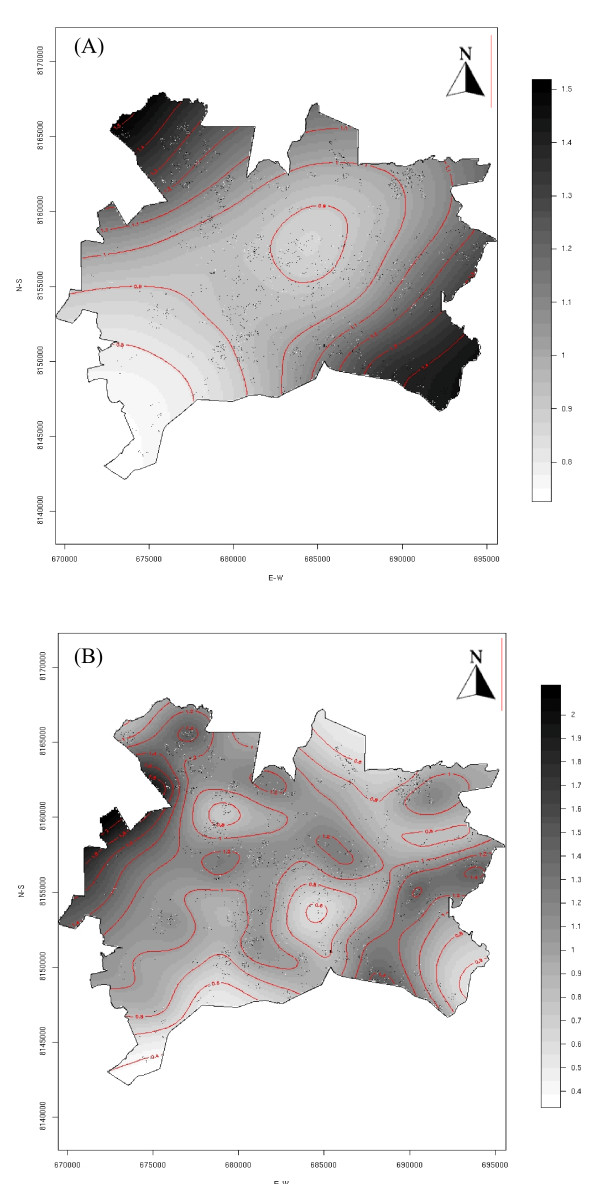
**Crude odds ratio using the GAM model.** (A) 2001 serosurvey; (B) 2002 serosurvey. Both surveys were conducted in the city of Goiania, Central Brazil.

**Figure 2:**
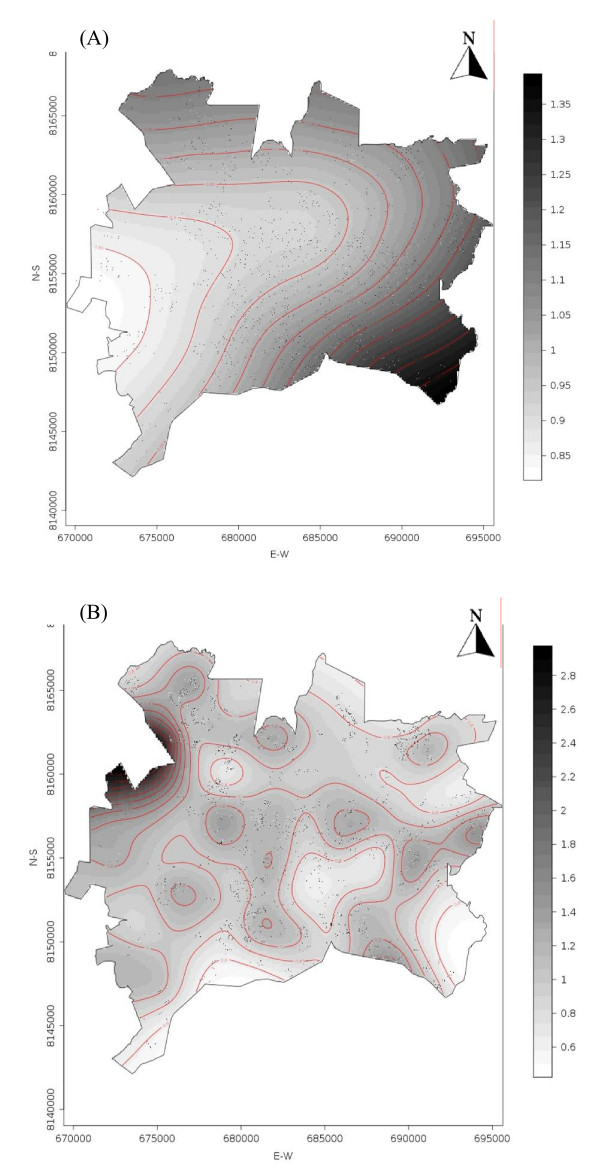
**Adjusted odds ratio for covariates: age group, sex and education using the GAM model. **(A) 2001 serosurvey; (B) 2002 serosurvey. Both surveys were conducted in the city of Goiania, Central Brazil.

Figure 1 and Figure 2 present the crude and adjusted odds ratio surfaces, using the GAM model. Crude and adjusted risk surface showed similar spatial features. For 2001, the spatial point analysis showed variation in risk surface with two hot spots identified in the southeast and northwest areas of the city; low risk areas were located in the central urban areas (Figure 1A). There seems to be a spread of infection from high to low prevalence areas detected in the 2001 survey.

In the 2002 survey, an increased risk was estimated for almost the entire city compared to the prior year. The central northern area of the city had a prevalence increase greater than 40%, corresponding to the higher incidence areas during the lag time between the surveys. In contrast, a less than 10% increase was observed in the central-southern region of the city during the study period.

## Discussion and conclusion

Our results showed a significant increase in dengue seroprevalence from 29.5 to 37.3% between two surveys conducted in the year 2001 and 2002 in a densely populated area where dengue virus had been recently introduced. Estimated risk in the second period of testing was higher in areas that previously had low prevalence when applying spatial analysis. Both GAM outputs, with and without covariates, showed similar risk areas, mainly located in the outskirts of the city in the year 2001 and further shifting to central areas as detected in the year 2002. The changes in the spatial distribution of prevalence in these consecutive years suggested the spread toward areas that previously had lower prevalence documented. This contrasts to previous high-risk areas where minor prevalence changes were observed. The herd immunity effect could explain the change of risk areas between serosurveys. In our setting, there was a tendency to increase dengue infection in areas considered of low prevalence in the first survey. The spatial variation in viral infection at population level (2001 versus 2002) was depicted by GAM maps.

The likely explanation for our findings is that individuals who became immune against one serotype previously are no longer at risk for the same serotype infection. These findings are in concordance with the established literature for the spread of infectious agents in general. In this study area, the increase in disease prevalence was probably due to the spread of DENV1, the predominant serotype according to laboratory surveillance since the virus introduction in the city in the year 1994 until the second survey [19].

It is worth mentioning that participants in both surveys were similar with respect to socio-demographic characteristics. Estimates relative risk by GAM that takes into account the dual outcome (infected and non infected participants) and also sociodemographic covariates had similar values. The advantage of the spatial point analysis presented was to show the uneven distribution of risk according to population density. Central areas with administrative buildings and the outskirts of city presented lower risks in both serosurveys. The initial limitation of comparing different point samplings was overcome by applying the GAM approach that consider the data set location with the advantage of taking into account the individual level co-variates [25,26]. Interestingly, the crude and adjusted maps in each year showed similar risk locations. Also the odds ratio calculated showed that low prevalence areas in the first survey were the major risk factor for increase in the odds ratio even taking into account potential confounders. Other study in Northeast Brazil pointed out the rapid spread of the virus during the first dengue epidemic in the city of Salvador applying Kernel method and R-Project computer software program to explore the spatial distribution of the reported cases[27]. Kernel based GAMs were also applied to produce risk maps of the spatial distribution of infant mortality and live-born as controls in a large city in Southeast Brazil [28].

In the current study, lower risk estimate from 2001 survey was one of the major predictors of subsequent risk in the next year. We also identified lower socioeconomic status, measured by educational history at the individual-level and income at the area-based level, as risk factors for disease risk in this setting. Individual or socio-economic variables that were previously associated with infection were also associated with these newly detected risk areas [10]. This association between poor living standards and dengue prevalence seems compatible with the distribution of notification of dengue cases by the official surveillance system in recent years. However, dengue cases are detected all over the city and not restricted to deprived areas.

One limitation of our study is the absence of household-specific data on the density of female mosquitoes. Infestation indices are used for routine surveillance in Brazil, despite the limited correlation between these indices and disease outbreaks [29]. Before the 2001 survey, approximately 40,000 dwellings were visited for an entomological assessment in the study area. The results from this vector investigation were available only aggregated by neighborhood, which are large administrative areas. This database was not incorporated in the analysis because it was not adequate to be linked to the dataset, using census-based data from small areas. Another limitation of our survey is that only ~5% of the participants were children (age 5–14 years old). The findings with increase seroprevalence with age may be explained to the longer period of viral exposure for older age-groups. Serosurveys conducted in a Northeast large city and other cities [8,30-33] have found similar results. Commercial EIA tests used in our population based survey, has also been applied in several other studies [34-36]. EIA test detects all four dengue serotypes (DENV1-4) according to the manufacturer with similar sensitivity and specificity to the standard reference test (hemaglutination inhibition test) [37,38].

Vector-borne infectious diseases such as dengue fever present complex and dynamic transmission patterns, which include vector density, human population density, herd immunity and circulating virus serotypes, and environmental conditions [7]. We are aware that to explain dengue spread by diffusion pattern may be a simplistic model. Several dengue episodes by different serotypes may occur in the same population, and there is a large range of factors in intra-urban area which may favor the maintenance of potential breeding sites [7]. Also climatologic factors such as temperature and rainfall variations between large regions [39-41] could influence spatial distribution. However, a previous investigation conducted in northern Brazil showed neither periodical nor significant correlation between meteorological variables and the pattern of dengue distribution [12]. Our setting is located in a savanna ecosystem in central Brazil, with little variability in temperature or rainfall observed in the inner city. Therefore, we believe that these climatologic factors are unlikely explanations to the observed spatial distribution of infected individuals within this urban setting in both surveys.

In our setting, consecutive household serosurveys were considered the first approach to evaluate the spatial distribution of infected individuals in the urban area. Although symptomatic dengue cases are officially registered by the Surveillance System, it is known that asymptomatic infectious outnumber the registered cases as reported in the 2001 survey [18]. Our findings are concordant with serosurvey from the southeast of Brazil, which showed that reported cases underestimate the number of infected individuals [42] and also in studies conducted in other countries [36]. In summary, we mapped areas with high vulnerability to dengue occurrence using point pattern location, avoiding the autocorrelation bias produced by administrative spatial unit. The spatial analytic approach of consecutive serosurveys provided insights into the distribution of dengue infection and could be informative for control interventions.

## Competing interests

The authors declare that they have no competing interests.

## Authors' contributions

JBSJ and IJM contributed equally to this work. JBSJ participated in the GPS/GIS data collection, data computing and the validation, the statistical analysis, drafted the manuscript and participated in the interpretation of data. IJM participated in the GPS/GIS data collection, in the clinical, biological data collection in the field site of Goiania, drafted the manuscript and in the interpretation of data. CB participated in the data computing, the statistical analysis and in the interpretation of data. WVS participated in the data computing, the statistical analysis and in the interpretation of data. MSC participated in the data computing, the statistical analysis and in the interpretation of data. NEN participated in the clinical, biological data collection in the field site of Goiania. RMO participated in the clinical, biological data collection in the field site of Goiania. OLMN participated in the GPS/GIS data collection, data computing and the validation CMTM the PI of the study led the team who conceived and design the studies. She participated in the data monitoring, QA/QC of the data, data analysis and correction of the manuscript. All authors read and approved the final manuscript.

## Pre-publication history

The pre-publication history for this paper can be accessed here:


